# Functional Knowledge of Pre-Exposure Prophylaxis for HIV Prevention Among Participants in a Web-Based Survey of Sexually Active Gay, Bisexual, and Other Men Who Have Sex With Men: Cross-Sectional Study

**DOI:** 10.2196/publichealth.8089

**Published:** 2018-01-23

**Authors:** Erin M Kahle, Stephen Sullivan, Rob Stephenson

**Affiliations:** ^1^ Center for Sexuality and Health Disparities School of Nursing University of Michigan Ann Arbor, MI United States

**Keywords:** human immunodeficiency virus, pre-exposure prophylaxis (PrEP), men who have sex with men

## Abstract

**Background:**

Awareness of pre-exposure prophylaxis (PrEP) for HIV prevention is increasing, but little is known about the functional knowledge of PrEP and its impact on willingness to use PrEP.

**Objective:**

The objective of this study was to assess the functional knowledge of PrEP among a sample of gay, bisexual, and other men who have sex with men (MSM) participating in a Web-based survey of sexually active MSM.

**Methods:**

Men at least 18 years old, residing in the United States, and reporting sex with a man in the previous 6 months were recruited through social networking websites. PrEP functional knowledge included the following 4 questions (1) efficacy of consistent PrEP use, (2) inconsistent PrEP use and effectiveness, (3) PrEP and condom use, and (4) effectiveness at reducing sexually transmitted infections (STIs). Ordinal logistic regression was used to identify respondent characteristics associated with PrEP functional knowledge. In a subsample of participants responding to HIV prevention questions, we compared willingness to use PrEP by response to PrEP functional knowledge using logistic regression analysis adjusted for age, race and ethnicity, and education level.

**Results:**

Among 573 respondents, PrEP knowledge was high regarding adherence (488/573, 85.2%), condom use (532/573, 92.8%), and STIs (480/573, 83.8%), but only 252/573 (44.0%) identified the correct efficacy. Lower functional PrEP knowledge was associated with minority race/ethnicity (*P*=.005), lower education (*P*=.01), and not having an HIV test in the past year (*P*=.02). Higher PrEP knowledge was associated with willingness to use PrEP (*P*=.009). Younger age was not associated with higher PrEP functional knowledge or willingness to use PrEP.

**Conclusions:**

PrEP knowledge was generally high in our study, including condom use and consistent use but may be lacking in higher risk MSM. The majority of respondents did not correctly identify PrEP efficacy with consistent use, which could impact motivation to seek out PrEP for HIV prevention. Targeted messaging to increase PrEP knowledge may increase PrEP use.

## Introduction

Since the release of clinical trials showing high efficacy of pre-exposure prophylaxis (PrEP) for preventing HIV acquisition [1-3] and the Food and Drug Administration (FDA) approval of Truvada for PrEP [4], increased evidence from PrEP demonstration projects show PrEP to be a robust addition to existing HIV prevention tools for reducing HIV acquisition in high-risk populations, such as sexually active men who have sex with men (MSM) [5-7]. Studies have found that consistent PrEP use is associated with high reduction in HIV acquisition, including reductions in HIV risk up to 92% among those with high adherence to daily pill regimen [2,8,9]. In addition, modeling data indicate increased PrEP coverage among MSM would result in a significant and sustained reduction in HIV prevalence in the United States [10]. The Centers for Disease Control and Prevention (CDC) have released guidelines for PrEP use, with specific recommendations for high-risk MSM [11].

Despite the proven efficacy of PrEP and recommendations for PrEP use among MSM, use of PrEP has remained low among priority populations at highest risk for HIV infection, including sexually active MSM [12-16]. In the past 5 years, awareness of PrEP for HIV prevention has been steadily increasing among MSM, and recent data indicate that the majority of MSM have heard of PrEP [13,15,17,18]. However, increased awareness of PrEP has not translated into high uptake of PrEP among MSM. Recent studies have found PrEP use at less than 10% among all MSM with lower rates among youth and racial/ethnic minority MSM [15,19-23]. Multiple individual barriers to scaling up PrEP have been identified, including concerns about adverse effects, cost, and stigma [12,19,24].

Considerable efforts are in progress to identify and reduce challenges in increasing PrEP coverage through targeted prevention messaging. However, although awareness of PrEP has been slowly but steadily increasing since the iPrEX study, a clinical trial finding significant PrEP efficacy among MSM [2], little is known about whether appropriate educational messaging about PrEP is reaching highest risk MSM. Low and inconsistent knowledge beyond general awareness of PrEP has been suggested as a potential barrier to PrEP uptake, but it has not been thoroughly assessed in relation to willingness to use PrEP [14,23,25,26]. Although there is increasing attention paid to the awareness of PrEP among MSM, there is relatively little attention paid to the presence of functional knowledge that is needed to use PrEP effectively. Functional knowledge includes knowledge around the efficacy of PrEP, the need to take PrEP consistently, the recommendation to use condoms while taking PrEP, and that PrEP does not reduce the transmission of sexually transmitted infections (STIs). Studies from other HIV prevention strategies, including male circumcision, condom use, and HIV testing, have found that level of knowledge is associated with increased willingness and uptake of these interventions [27-29], suggesting that knowledge beyond basic awareness of PrEP could impact willingness to use PrEP. Although understanding the proportion of MSM who have heard of PrEP will likely inform an understanding of awareness, an assessment of the levels of functional knowledge may have more utility for understanding whether PrEP messaging is being absorbed by MSM. Thus, we sought to assess functional knowledge of PrEP, including efficacy, consistency, impact on STIs, and condom use, among a Web-based sample of predominantly white, college educated, sexually active MSM participating in a Web-based survey of HIV knowledge and priorities.

## Methods

### Study Population

The Prioritizing U survey was conducted in August and September 2015 to collect cross-sectional, self-reported data on HIV knowledge, prevention, and priorities among gay, bisexual, and other MSM. The survey and data collection methods have been previously described [30]. Briefly, study participants were recruited through convenience sampling methods using We-based banner advertisements posted on social media targeting user profiles matching the study eligibility criteria. Men who clicked on the Webpage link were directed to an introductory page and given a brief screening questionnaire. Men were eligible for the survey if they reported being 18 years of age or older, identified as male, resided in the United States, and reported sex with a man in the past 6 months. Participants who completed the consent page and were eligible to participate completed a self-administered, confidential Web-based survey. In total, 2241 men were screened eligible and completed the survey. The study was approved by the University of Michigan Institutional Review Board. No monetary incentives were provided to the participants.

**Figure 1:**
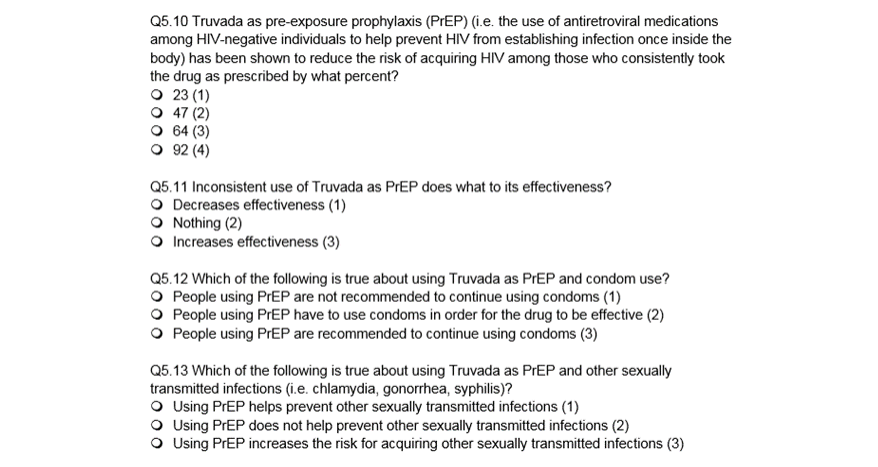
Pre-exposure prophylaxis (PrEP) functional knowledge questions, Prioritizing U, 2015.

The survey included questions on demographics (eg, age, race and ethnicity, education, income, and employment), sexual behavior with male and female partners in the past 3 months, and HIV testing history. Participants were also asked a series of multiple-choice knowledge questions about HIV infection and prevention developed by the research team and adapted from previous surveys conducted in multiple populations [31,32], including questions from the stable and internally consistent HIV Knowledge Scale [33]. The HIV knowledge questions also included the following 4 questions about PrEP: (1) percent reduction in acquiring HIV through consistent use of PrEP, (2) decreased effectiveness of PrEP with inconsistent use, (3) continued use of condoms recommended for people using PrEP, and (4) PrEP does not help prevent other STIs (Figure 1). Additionally, participants were given a list of HIV prevention methods, including PrEP, and asked if they knew about, have used, or would use each method. However, substantially fewer participants responded to the HIV prevention questions. The prevention questions were presented at the end of the survey, and although we do not have information to explain the low response for these questions, survey fatigue or survey formatting could be possible reasons that participants did not complete the full survey. We wanted to assess the relationship between functional PrEP knowledge and willingness to use PrEP; thus, all analyses of willingness to use PrEP included only those respondents that responded to the HIV prevention questions.

### Statistical Analysis

For this analysis, we included only participants who self-reported HIV status as negative or unknown, had anal sex with a male partner in the past 3 months, and responded to PrEP knowledge questions. We excluded participants that did not provide their actual age (the screened questionnaire only confirmed age ≥18 years old). The smaller subset that completed the HIV prevention questions was compared with the larger cohort using chi-square test statistics. PrEP knowledge was defined as the proportion of each PrEP question correctly answered and an ordinal PrEP score calculated by the total number of correct responses.

We assessed PrEP knowledge by demographic characteristics (eg, age, race and ethnicity, education, and geographic region), sexual risk behavior in the past 3 months (eg, condomless sex, multiple male sex partners, and HIV status of primary male sex partner), HIV testing history (eg, ever tested and tested in the past 12 months), and interest in using PrEP (eg, have used and would use). Associations with PrEP knowledge were measured for the full sample and separately for younger respondents, aged 18-29 years, as this population is at particularly high risk for HIV acquisition with lower PrEP uptake. We assessed individual respondent characteristics to determine individual associations with PrEP knowledge. Predictors found to be significantly associated with PrEP knowledge at the *P*<.05 level in univariate analyses were assessed in 2 multiple ordinal regression models with tests for proportional odds. The models were not found to violate the proportional odds assumption. The regression coefficients in the multiple ordinal regression models were used to examine the log-odds of higher PrEP knowledge score with results expressed in terms of cumulative adjusted odds ratios (adjOR) with 95% CIs. To assess willingness to use PrEP by PrEP knowledge score, we included only participants that responded to the HIV prevention questions, including willingness to use PrEP. We used a logistic regression model adjusted for statistically significant predictors from univariate analyses. We assessed the relationship between perceived efficacy and willingness to use PrEP using a Cochran-Armitage test for trend comparing responses to the efficacy knowledge question by PrEP willingness. All analyses were conducted in Statistical Analysis Software (SAS) version 9.4 (Cary, NC).

## Results

Among 2241 eligible participants with complete surveys, 80 (3.60%) were excluded for not providing their age. Of the remaining, 2012 (93.10%) reported negative or unknown/never tested HIV status, of which 970 (48.21%) reported at least 1 anal sex partner in the previous 3 months. Moreover, 573 out of 970 (59.1%) completed all PrEP knowledge questions and were included in this analysis. The median age of the study participants was 43 years (range 18-86), with the majority (375/573, 65.4%) 35 years or older (Table 1). Most participants were white (80.5%), college educated (58.6%), and employed full-time (77.5%). Study participants reported an average of 3 (range 1-200) anal sex partners in the past 3 months, and most (71.9%) had anal sex without a condom at least once. Current PrEP use was reported among 11 (2.1%) participants.

Nearly half of the participants (280/573, 48.9%) responded to HIV prevention questions (Table 1). Compared with participants that did not respond to the HIV prevention questions, those that responded to the HIV prevention questions were more likely to be younger (<35 years, 41.4% vs 28.0%, *P*<.001), from the South (42.1% vs 30.0%, *P*=.003), had multiple anal sex partners in the past year (48.9% vs 35.8%, *P*=.02), and have been HIV tested in the previous year (54.3% vs 49.2%, *P*=.004).

**Table 1:** Characteristics of respondents, Prioritizing U survey, 2015.

Characteristics		PrEP^a^ knowledge question respondents (N=573) n (%)	Prevention questions respondents (N=280) n (%)
**Age (years)**			
	18-24	101 (17.6)	62 (22.1)
	25-34	97 (16.9)	54 (19.3)
	35-44	71 (12.4)	32 (11.4)
	45+	304 (53.1)	132 (47.1)
**Race**			
	Black/African American	12 (2.1)	7 (2.5)
	Hispanic/Latino	68 (11.9)	34 (12.1)
	White	461 (80.5)	224 (80.0)
	Other/multiple	31 (5.4)	15 (5.4)
**Geographic region**			
	Midwest	139 (24.3)	64 (22.9)
	Northeast	105 (18.3)	40 (14.3)
	South	206 (36.0)	118 (42.1)
	West	112 (19.6)	53 (18.9)
**Education**			
	<High school or diploma/equivalent	40 (7.0)	15 (5.4)
	Some college or technical degree	197 (34.4)	97 (34.6)
	College degree or postgraduate	336 (58.6)	168 (60.0)
**Employment**			
	Full-time work	444 (77.5)	208 (74.3)
	Part-time work	62 (10.8)	35 (12.50
	Unemployed/other	66 (11.5)	37 (13.2)
**Number of anal sex partners in the past 3 months**			
	1	331 (57.8)	143 (51.1)
	2-4	157 (27.4)	83 (29.6)
	5+	85 (14.8)	54 (19.3)
**Any condomless anal sex in the past 3 months**			
	Yes	412 (71.9)	210 (75.0
	No	126 (22.0)	55 (19.6)
**Condomless anal sex with nonprimary partner in the past 3 months**			
	Yes	158 (27.6)	102 (36.4)
	No	415 (72.4)	178 (63.6)
**More recent HIVtest**			
	Within the past year	315 (55.0)	171 (61.1)
	>1 year ago	198 (34.6)	85 (30.4)
	Never/not sure when last tested	60 (10.5)	24 (8.6)

^a^PrEP: pre-exposure prophylaxis.

**Figure 2:**
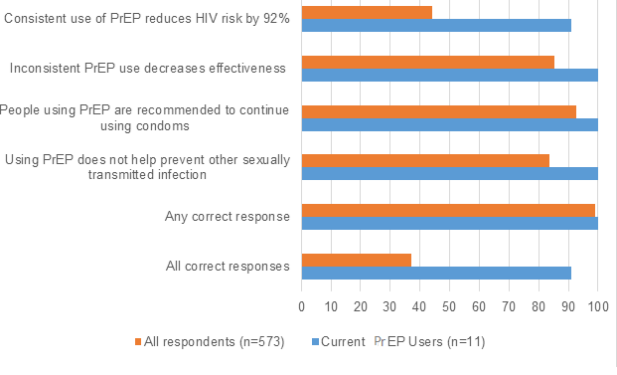
The percentage distribution of correct responses to pre-exposure prophylaxis (PrEP) functional knowledge questions by all respondents and respondents reporting current PrEP use, Prioritizing U, 2015. The percentage distribution of correct responses by full sample of respondents (orange) and by respondents reporting current PrEP use (blue), and percentage of correct responses for the four PrEP knowledge questions, percentage with any of the four PrEP knowledge questions, and percentage of respondents with correct responses for all four PrEP knowledge questions.

The majority of respondents identified the correct response to 3 of the 4 PrEP knowledge questions: (1) inconsistent PrEP use decreases effectiveness (85.2%), (2) people using PrEP are recommended to continue using condoms (92.8%), and (3) PrEP does not prevent other STIs (83.8%). Less than half of the respondents correctly identified the percent reduction in HIV risk with consistent PrEP use (252/573, 44.0%). Overall, 213 out of 573 (37.2%) correctly answered all 4 PrEP knowledge questions, 224 out of 573 (39.1%) answered only 3 questions correctly, 98 out of 573 (17.1%) answered only 2 correctly, 32 out of 573 (5.6%) answered only 1 correctly, and 6 out of 573 (1.1%) answered none of the questions correctly. Nearly all current PrEP users (90.9%) answered all questions correctly (Figure 2).

All responses for PrEP knowledge questions are shown in Table 2. Higher PrEP knowledge scores were found to be significantly associated with having at least some college education (adjOR 3.11, 95% CI 1.71-5.65, *P*<.001), having an HIV test in the past year (adjOR 2.14, 95% CI 1.54-2.98, *P* ≤.001), and reporting condomless sex with a nonprimary partner in the past 3 months (adjOR 2.83, 95% CI 1.71-4.68, *P*<.001). Respondents reporting nonwhite race/ethnicity were significantly more likely to have lower PrEP knowledge scores (adjOR 0.56, 95% CI 0.34-0.84, *P*=.005). Age, geographic region of residence, and number of male sex partners were not significantly associated with correctly responding to PrEP knowledge questions. Among only younger respondents, lower PrEP knowledge was only found to be associated with nonwhite race/ethnicity (adjOR 0.26, 95% CI 0.10-0.71, *P*=.01).

Participants that responded to HIV prevention questions had a significantly higher odds of correctly responding to PrEP knowledge questions (adjOR 3.38, 95% CI 2.44-4.69, *P*<.001). Willingness to use PrEP was significantly associated with correct responses to PrEP knowledge questions (adjOR 1.62, 95% CI 1.13-2.33, *P*=.009). Specifically, correctly identifying PrEP efficacy was significantly associated with those that would use PrEP (adjOR 2.65, 95% CI 1.55-4.54 *P*<.001; Table 3). Furthermore, we found a significant trend in willingness to use PrEP by the level of efficacy selected by respondents, with those that perceived the lowest efficacy reporting lower willingness to use PrEP (chi-square *P*<.001). Younger respondents were not significantly more likely to report greater willingness to use PrEP. Among younger respondents, willingness to use PrEP was associated with correct response to the PrEP efficacy question (adjOR 4.95, 95% CI 1.63-15.08, *P*=.003) and correctly responding to all PrEP knowledge questions (adjOR 3.48, 95% CI 1.14-10.64, *P*=.02).

**Table 2:** Distribution of responses for pre-exposure prophylaxis functional knowledge questions for all respondents and younger respondents (age 18-29 years), Prioritizing U survey, 2015.

Questions		All respondents (N=573) n (%)	Younger^a^ respondents only (N=153) n (%)
**Consistent use of PrEP**^b^ **reduces HIV risk by what percent?**			
	23	113 (19.7)	23 (15.0)
	47	116 (20.2)	42 (27.5)
	64	92 (16.1)	30 (19.6)
	92^d^	252 (44.0)	58 (37.9)
**Inconsistent use of PrEP does what to its effectiveness?**			
	Decreases effectiveness^c^	488 (85.2)	130 (85.0)
	Nothing	44 (7.7)	11 (7.2)
	Increases effectiveness	41 (7.2)	12 (7.8)
**Which of the following is true about using PrEP and condom use?**			
	Not recommended to continue using condoms	13 (2.3)	5 (3.3)
	Condoms are required for PrEP to be effective	28 (5.0)	10 (6.5)
	Recommended to continue using condoms^c^	532 (92.8)	138 (90.2)
**Which of the following is true about using PrEP and other STIs**^d^**?**			
	PrEP helps prevent other STIs	74 (12.91)	23 (15.03)
	Does not help prevent other STIs^c^	480 (83.8)	125 (81.7)
	Increases risk of STIs	19 (3.3)	5 (3.3)

^a^Age 18-29 years.

^b^PrEP: pre-exposure prophylaxis.

^c^Correct response.

^d^STIs: sexually transmitted infections.

**Table 3:** Association between correct pre-exposure prophylaxis (PrEP) functional knowledge responses and willingness to use PrEP by each PrEP functional knowledge question and correct responses to all PrEP knowledge questions, Prioritizing U survey, 2015.

Questions		Would use PrEP^a^, n (%)		adjOR^b^ (95% CI)^c^
		Yes (N=204)	No (N=80)	
**Consistent use of PrEP reduces HIVrisk by 92%**				
	Correct	126 (61.8)	31 (38.8	2.65 (1.55-4.54)
	Incorrect	78 (38.2)	49 (61.3)	
**Inconsistent PrEP use decreases effectiveness**				
	Correct	183 (90.2)	70 (87.5)	0.52 (0.07-3.91)
	Incorrect	20 (9.9)	10 (12.5)	
**People using PrEP are recommended to continue using condoms**				
	Correct	200 (98.5)	78 (97.5)	1.50 (0.07-31.06)
	Incorrect	3 (1.5)	2 (2.5)	
**Using PrEP does not help prevent other STIs**^d^				
	Correct	184 (92.0)	73 (91.3)	1.55 (0.37-6.45)
	Incorrect	16 (8.0)	7 (8.8)	
**Responded correctly to all PrEP knowledge questions**				
	Correct	106 (52.0)	27 (33.8)	3.48 (1.14-10.64)
	Incorrect	98 (48.0)	53 (66.3)	

^a^PrEP: pre-exposure prophylaxis.

^b^adjOR: adjusted odds ratio.

^c^adjusted for age, race/ethnicity, and education

^d^STIs: sexually transmitted infections

## Discussion

### Principal Findings

Understanding the role of PrEP in HIV prevention beyond simple awareness may be critical in making informed decisions to use PrEP. In our analysis, we found that higher functional knowledge was associated with willingness to use PrEP among a sample of MSM participating in a Web-based survey of HIV knowledge and priorities. We found respondents to be generally knowledgeable about PrEP, although knowledge was higher among white, college-educated MSM. Lower awareness of PrEP has been found, in previous studies, to be associated with younger age, lower education, and racial/ethnic minorities [21,34] and is consistent with our findings of lower knowledge of PrEP among racial/ethnic minority and lower-educated MSM. Additionally, we found PrEP knowledge to be lower among those who had not had an HIV test in the past year, suggesting a lack of opportunity to learn more about PrEP through routine testing and counseling. Studies have found lower PrEP knowledge among primary care physicians compared with HIV specialists that may limit opportunities for MSM to learn about and receive PrEP, particularly among MSM not already engaged in HIV prevention [35-39].

Perceptions of PrEP effectiveness and understanding of how PrEP fits into sexual risk behavior is necessary to increase interest in using PrEP. In general, the majority of respondents successfully answered 3 of the 4 PrEP knowledge questions: inconsistent PrEP use decreases effectiveness in preventing HIV, continued condom use is recommended when using PrEP, and PrEP does not prevent other STIs. However, less than half of the respondents correctly selected the correct percent reduction in HIV risk with consistent PrEP use. Communicating information about PrEP is challenging due to the ongoing studies exploring alternative regimens and drug options. The effectiveness of PrEP among MSM may be the most difficult to articulate in HIV prevention messaging due to the seemingly discrepant results from PrEP clinical trials and demonstration projects. Efficacy results from PrEP clinical trials ranged from futility to 75%, with additional results as high as 92% in iPrEX among adherent participants [40]. Furthermore, the misinformation and misinterpretation of efficacy may lead to lower understanding of individual PrEP effectiveness [26,41]. The low proportion of correct responses to efficacy with consistent PrEP use may be the result of the varying data and interpretations of findings from multiple PrEP studies. We found that perceptions of PrEP efficacy were associated with willingness to use PrEP, suggesting a critical importance of effectively messaging PrEP efficacy to increase uptake.

We found that most respondents knew the importance of using PrEP consistently and continued condom use with PrEP based on CDC recommendations [11]. However, it is not clear if these recommendations are translated into practice among MSM using PrEP. Adherence to daily PrEP regimens is a primary concern of health care providers [42-44], and low retention in PrEP programs is associated with HIV acquisition [45]. Results from iPrEX and other clinical trials showed a dramatic decrease in PrEP efficacy with adherence of less than 80% across all populations, leading to guidelines for PrEP for HIV prevention in addition to other prevention methods [2,11]. Furthermore, data from recent studies show an increase in condomless sex among MSM since PrEP implementation was initiated, particularly among MSM using PrEP, although it is unclear if this trend is the direct result of the introduction of PrEP [13,46]. We found higher sexual risk, measured by condomless sex with a nonprimary male partner, to be associated with higher PrEP knowledge. If higher knowledge is consistent with increased PrEP use, then our findings are consistent with other studies showing higher PrEP use among MSM engaged in higher risk sexual activity and higher perception of HIV risk [13,47,48]. In our subgroup analysis, willingness to use PrEP was significantly associated with higher PrEP knowledge, although the sample was too small to make adequate comparisons by sexual risk behavior. This finding is important in illustrating the need of adequate and appropriate communication of PrEP information to high-risk MSM.

Participants in our study were generally aware of the need for continued condom use and that PrEP does not decrease risk of STIs. Increases in STIs have recently been noted among MSM PrEP users in clinical trials and PrEP demonstration projects, indicating potential risk compensation associated with PrEP use through reduction in condom usage [49,50]. However, in a recent systematic review, Freeborn and Portillo did not find conclusive data that PrEP users engage in increased sexual risk behavior and rates of STIs did not significantly increase [51]. Furthermore, the benefit of PrEP in HIV risk reduction seems to outweigh moderate increases in risk compensation among MSM [52]. PrEP should continue to be seen as a complementary risk reduction tool along with condoms and routine HIV testing.

Willingness to use PrEP was significantly associated with higher PrEP functional knowledge among MSM participating in our study. This is consistent with previous studies finding an association between PrEP awareness and willingness to use PrEP [53]. However, PrEP awareness and knowledge is a single factor in a host of indicators for PrEP use, and increasing functional knowledge about PrEP is not a sufficient strategy for increasing PrEP uptake among MSM [54]. Unwillingness to use PrEP has been found to be associated with concerns about side effects, access to health care, and HIV-related stigma across multiple populations of MSM [19,20,55-57]. Addressing concerns about PrEP and reducing barriers to PrEP access may be more critical to motivating PrEP, particularly in populations where general PrEP awareness is already high.

### Limitations

We acknowledge several limitations to our study. First, we used a convenience sampling method with recruitment through online social media; thus, our sample is not representative of MSM or specifically high-risk MSM. Our sample was older, primarily white and college educated and does not reflect the highest risk MSM, specifically young and race/ethnic minority MSM. PrEP awareness and use have been shown to be higher among older white MSM [21,23,58], and our study shows lower PrEP knowledge among nonwhite and lower educated MSM, suggesting a need for targeted messaging to highest risk MSM. However, we found no significant difference in PrEP functional knowledge by age, and younger respondents reported a higher proportion of willingness to use PrEP. Further research is needed to understand perceptions and interpretations of PrEP information among young and racial/ethnic minority MSM. Second, our analysis of willingness to use PrEP was limited to a subgroup of respondents that answered questions about HIV prevention. We do not have information to explain the substantial drop in responses to the HIV prevention questions, although the survey was long and participants may have felt survey fatigue by the time they were presented with these questions. Thus, due to smaller sample sizes, we were not able to make more detailed comparisons among MSM willing and not willing to use PrEP. Third, we did not collect information on where respondents heard/learned information about PrEP that would be useful in exploring opportunities for increasing messaging about PrEP and HIV prevention to MSM. Finally, although we tested the survey with a panel of volunteers, we do not have data to determine if the PrEP knowledge questions were fully understood by the study participants, particularly the PrEP efficacy question. We recognize that the questions may have been more challenging to participants with little or no prior knowledge about PrEP and recommend additional testing of these questions before use in future assessments of PrEP knowledge.

### Conclusions

Despite these limitations, our findings provide additional information to increasing data on PrEP perceptions and intentions among MSM. Prioritization of PrEP to highest risk populations optimizes the impact of PrEP in reducing HIV infections [6,47,59]. However, to increase PrEP coverage, it is imperative to increase knowledge and acceptance of PrEP among targeted populations through increased education and messaging at the individual, provider, and community level [60,61]. Furthermore, additional research is needed to create more effective messaging tools for increasing PrEP knowledge and acceptability among MSM through community outreach, public health campaigns, and provider participation [54,61]. Our findings show that PrEP functional knowledge is generally high, although not consistent across all demographics of MSM. More data are needed to determine if PrEP knowledge translates to motivation to use and retention in PrEP.

## Abbreviations

adjOR: adjusted odds ratios
CDC: Centers for Disease Control and Prevention
FDA: Food and Drug Administration
MSM: men who have sex with men
PrEP: pre-exposure prophylaxis
STIs: sexually transmitted infections

## References

[1] Baeten JM, Donnell D, Ndase P, Mugo NR, Campbell JD, Wangisi J, Tappero JW, Bukusi EA, Cohen CR, Katabira E, Ronald A, Tumwesigye E, Were E, Fife KH, Kiarie J, Farquhar C, John-Stewart G, Kakia A, Odoyo J, Mucunguzi A, Nakku-Joloba E, Twesigye R, Ngure K, Apaka C, Tamooh H, Gabona F, Mujugira A, Panteleeff D, Thomas KK, Kidoguchi L, Krows M, Revall J, Morrison S, Haugen H, Emmanuel-Ogier M, Ondrejcek L, Coombs RW, Frenkel L, Hendrix C, Bumpus NN, Bangsberg D, Haberer JE, Stevens WS, Lingappa JR, Celum C, Partners PST (2012). Antiretroviral prophylaxis for HIV prevention in heterosexual men and women. N Engl J Med.

[2] Grant RM, Lama JR, Anderson PL, McMahan V, Liu AY, Vargas L, Goicochea P, Casapía M, Guanira-Carranza JV, Ramirez-Cardich ME, Montoya-Herrera O, Fernández T, Veloso VG, Buchbinder SP, Chariyalertsak S, Schechter M, Bekker L, Mayer KH, Kallás EG, Amico KR, Mulligan K, Bushman LR, Hance RJ, Ganoza C, Defechereux P, Postle B, Wang F, McConnell JJ, Zheng J, Lee J, Rooney JF, Jaffe HS, Martinez AI, Burns DN, Glidden DV, iPrEx ST (2010). Preexposure chemoprophylaxis for HIV prevention in men who have sex with men. N Engl J Med.

[3] Thigpen MC, Kebaabetswe PM, Paxton LA, Smith DK, Rose CE, Segolodi TM, Henderson FL, Pathak SR, Soud FA, Chillag KL, Mutanhaurwa R, Chirwa LI, Kasonde M, Abebe D, Buliva E, Gvetadze RJ, Johnson S, Sukalac T, Thomas VT, Hart C, Johnson JA, Malotte CK, Hendrix CW, Brooks JT, TDF2 SG (2012). Antiretroviral preexposure prophylaxis for heterosexual HIV transmission in Botswana. N Engl J Med.

[4] United States Food and Drug Administration https://aidsinfo.nih.gov/news/1254/fda-approves-first-drug-for-reducing-the-risk-of-sexually-acquired-hiv-infection.

[5] Liu AY, Cohen SE, Vittinghoff E, Anderson PL, Doblecki-Lewis S, Bacon O, Chege W, Postle BS, Matheson T, Amico KR, Liegler T, Rawlings MK, Trainor N, Blue RW, Estrada Y, Coleman ME, Cardenas G, Feaster DJ, Grant R, Philip SS, Elion R, Buchbinder S, Kolber MA (2016). Preexposure prophylaxis for HIV infection integrated with municipal- and community-based sexual health services. J Am Med Assoc Intern Med.

[6] Smith DK, Van Handel M, Wolitski RJ, Stryker JE, Hall HI, Prejean J, Koenig LJ, Valleroy LA (2015). Vital signs: estimated percentages and numbers of adults with indications for preexposure prophylaxis to prevent HIV acquisition--United States, 2015. J Miss State Med Assoc.

[7] Wheeler DP, Nelson LE, Wilton L, Hightow-Weidman L, Shoptaw S, Magnus M, Beauchamp G, Watkins P, Mayer KH (2016). HPTN 073: PrEP uptake and use by black men who have sex with men in 3 US cities. http://www.croiconference.org/sites/default/files/posters-2016/883LB.pdf.

[8] Donnell D, Baeten JM, Bumpus NN, Brantley J, Bangsberg DR, Haberer JE, Mujugira A, Mugo N, Ndase P, Hendrix C, Celum C (2014). HIV protective efficacy and correlates of tenofovir blood concentrations in a clinical trial of PrEP for HIV prevention. J Acquir Immune Defic Syndr.

[9] Kashuba AD, Patterson KB, Dumond JB, Cohen MS (2012). Pre-exposure prophylaxis for HIV prevention: how to predict success. Lancet.

[10] Jenness SM, Goodreau SM, Rosenberg E, Beylerian EN, Hoover KW, Smith DK, Sullivan P (2016). Impact of the Centers for Disease Control's HIV preexposure prophylaxis guidelines for men who have sex with men in the United States. J Infect Dis.

[11] Centers for Disease Control and Prevention https://www.cdc.gov/hiv/pdf/prepguidelines2014.pdf.

[12] King HL, Keller SB, Giancola MA, Rodriguez DA, Chau JJ, Young JA, Little SJ, Smith DM (2014). Pre-exposure prophylaxis accessibility research and evaluation (PrEPARE Study). AIDS Behav.

[13] Strauss BB, Greene GJ, Phillips G, 2nd, Bhatia R, Madkins K, Parsons JT, Mustanski B (2017). Exploring patterns of awareness and use of HIV pre-exposure prophylaxis among young men who have sex with men. AIDS Behav.

[14] Rucinski KB, Mensah NP, Sepkowitz KA, Cutler BH, Sweeney MM, Myers JE (2013). Knowledge and use of pre-exposure prophylaxis among an online sample of young men who have sex with men in New York City. AIDS Behav.

[15] Hoots BE, Finlayson T, Nerlander L, Paz-Bailey G, National HIV Behavioral Surveillance Study Group (2016). Willingness to take, use of, and indications for pre-exposure prophylaxis among men who have sex with men-20 US cities, 2014. Clin Infect Dis.

[16] Hamel LF, Hoff T, Kates J, Levine S, Dawson L, Kaiser Family Foundation http://files.kff.org/attachment/survey-hivaids-in-the-lives-of-gay-and-bisexual-men-in-the-united-states.

[17] Goedel WC, Halkitis PN, Greene RE, Hickson DA, Duncan DT (2016). HIV risk behaviors, perceptions, and testing and preexposure prophylaxis (PrEP) awareness/use in grindr-using men who have sex with men in Atlanta, Georgia. J Assoc Nurses AIDS Care.

[18] Flash C, Landovitz R, Giler RM, Ng L, Magnuson D, Wooley SB, Rawlings K (2014). Two years of Truvada for pre-exposure prophylaxis utilization in the US. J Int AIDS Soc.

[19] Goedel WC, Halkitis PN, Greene RE, Duncan DT (2016). Correlates of awareness of and willingness to use pre-exposure prophylaxis (PrEP) in gay, bisexual, and other men who have sex with men who use geosocial-networking smartphone applications in New York City. AIDS Behav.

[20] Kelley CF, Kahle E, Siegler A, Sanchez T, Del Rio C, Sullivan PS, Rosenberg ES (2015). Applying a PrEP continuum of care for men who have sex with men in Atlanta, Georgia. Clin Infect Dis.

[21] Eaton LA, Driffin DD, Bauermeister J, Smith H, Conway-Washington C (2015). Minimal awareness and stalled uptake of pre-exposure prophylaxis (PrEP) among at risk, HIV-negative, black men who have sex with men. AIDS Patient Care STDS.

[22] Brooks RA, Landovitz RJ, Regan R, Lee S, Allen Jr VC (2015). Perceptions of and intentions to adopt HIV pre-exposure prophylaxis among black men who have sex with men in Los Angeles. Int J STD AIDS.

[23] Bauermeister JA, Meanley S, Pingel E, Soler JH, Harper GW (2013). PrEP awareness and perceived barriers among single young men who have sex with men. Curr HIV Res.

[24] Eaton LA, Driffin DD, Smith H, Conway-Washington C, White D, Cherry C (2014). Psychosocial factors related to willingness to use pre-exposure prophylaxis for HIV prevention among black men who have sex with men attending a community event. Sex Health.

[25] Dolezal C, Frasca T, Giguere R, Ibitoye M, Cranston RD, Febo I, Mayer KH, McGowan I, Carballo-Diéguez A (2015). Awareness of post-exposure prophylaxis (PEP) and pre-exposure prophylaxis (prep) is low but interest is high among men engaging in condomless anal sex with men in Boston, Pittsburgh, and San Juan. AIDS Educ Prev.

[26] Kurtz SP, Buttram ME (2016). Misunderstanding of pre-exposure prophylaxis use among men who have sex with men: public health and policy implications. LGBT Health.

[27] Zhou B, Ning C, McCann CD, Liao Y, Yang X, Zou Y, Jiang J, Liang B, Abdullah AS, Qin B, Upur H, Zhong C, Ye L, Liang H (2017). Impact of educational interventions on acceptance and uptake of male circumcision in the general population of Western China: a multicenter cohort study. Sci Rep.

[28] Lammers J, van Wijnbergen SJ, Willebrands D (2013). Condom use, risk perception, and HIV knowledge: a comparison across sexes in Nigeria. HIV AIDS (Auckl).

[29] Adeneye AK, Brieger WR, Mafe MA, Adeneye AA, Salami KK, Titiloye MA, Adewole TA, Agomo PU (2006). Willingness to seek HIV testing and counseling among pregnant women attending antenatal clinics in Ogun State, Nigeria. Int Q Community Health Educ.

[30] Sullivan S, Stephenson R (2017). Perceived HIV prevalence accuracy and sexual risk behavior among gay, bisexual, and other men who have sex with men in the United States. AIDS Behav.

[31] Wagenaar BH, Sullivan PS, Stephenson R (2012). HIV knowledge and associated factors among internet-using men who have sex with men (MSM) in South Africa and the United States. PLoS One.

[32] Grin B, Chan PA, Operario D (2013). Knowledge of acute human immunodeficiency virus infection among gay and bisexual male college students. J Am Coll Health.

[33] Carey MP, Schroder KEE (2002). Development and psychometric evaluation of the brief HIV Knowledge Questionnaire. AIDS Educ Prev.

[34] Misra K, Udeagu C (2017). Disparities in awareness of HIV postexposure and preexposure prophylaxis among notified partners of HIV-positive individuals, New York City 2015-2017. J Acquir Immune Defic Syndr.

[35] Petroll AE, Walsh JL, Owczarzak JL, McAuliffe TL, Bogart LM, Kelly JA (2017). PrEP awareness, familiarity, comfort, and prescribing experience among US primary care providers and HIV specialists. AIDS Behav.

[36] Blackstock OJ, Moore BA, Berkenblit GV, Calabrese SK, Cunningham CO, Fiellin DA, Patel VV, Phillips KA, Tetrault JM, Shah M, Edelman EJ (2017). A cross-sectional online survey of HIV pre-exposure prophylaxis adoption among primary care physicians. J Gen Intern Med.

[37] Hakre S, Blaylock JM, Dawson P, Beckett C, Garges EC, Michael NL, Danaher PJ, Scott PT, Okulicz JF (2016). Knowledge, attitudes, and beliefs about HIV pre-exposure prophylaxis among US Air Force Health Care Providers. Medicine (Baltimore).

[38] Clement ME, Seidelman J, Wu J, Alexis K, McGee K, Okeke NL, Samsa G, McKellar M (2017). An educational initiative in response to identified PrEP prescribing needs among PCPs in the Southern U.S. AIDS Care.

[39] Smith DK, Mendoza MC, Stryker JE, Rose CE (2016). PrEP awareness and attitudes in a national survey of primary care clinicians in the United States, 2009-2015. PloS One.

[40] Baeten J, Celum C (2013). HIV Prevention at CROI 2013. http://www.natap.org/2013/CROI/croi_75.htm.

[41] Underhill K, Morrow KM, Colleran C, Calabrese SK, Operario D, Salovey P, Mayer KH (2016). Explaining the efficacy of pre-exposure prophylaxis (PrEP) for HIV prevention: a qualitative study of message framing and messaging preferences among US men who have sex with men. AIDS Behav.

[42] Krakower D, Ware N, Mitty JA, Maloney K, Mayer KH (2014). HIV providers' perceived barriers and facilitators to implementing pre-exposure prophylaxis in care settings: a qualitative study. AIDS Behav.

[43] Tripathi A, Ogbuanu C, Monger M, Gibson JJ, Duffus WA (2012). Preexposure prophylaxis for HIV infection: healthcare providers' knowledge, perception, and willingness to adopt future implementation in the southern US. South Med J.

[44] Desai M, Gafos M, Dolling D, McCormack S, Nardone A, PROUD study (2016). Healthcare providers' knowledge of, attitudes to and practice of pre-exposure prophylaxis for HIV infection. HIV Med.

[45] Chan PA, Mena L, Patel R, Oldenburg CE, Beauchamps L, Perez-Brumer AG, Parker S, Mayer KH, Mimiaga MJ, Nunn A (2016). Retention in care outcomes for HIV pre-exposure prophylaxis implementation programmes among men who have sex with men in three US cities. J Int AIDS Soc.

[46] Chen YH, Snowden JM, McFarland W, Raymond HF (2016). Pre-exposure prophylaxis (PrEP) use, seroadaptation, and sexual behavior among men who have sex with men, San Francisco, 2004-2014. AIDS Behav.

[47] Kessler J, Myers JE, Nucifora KA, Mensah N, Toohey C, Khademi A, Cutler B, Braithwaite RS (2014). Evaluating the impact of prioritization of antiretroviral pre-exposure prophylaxis (PrEP) in New York City. AIDS.

[48] Gamarel KE, Golub SA (2015). Intimacy motivations and pre-exposure prophylaxis (PrEP) adoption intentions among HIV-negative men who have sex with men (MSM) in romantic relationships. Ann Behav Med.

[49] Kojima N, Davey DJ, Klausner JD (2016). Pre-exposure prophylaxis for human immunodeficiency virus and sexually transmitted infection acquisition among men who have sex with men. AIDS.

[50] Scott HM, Klausner JD (2016). Sexually transmitted infections and pre-exposure prophylaxis: challenges and opportunities among men who have sex with men in the US. AIDS Res Ther.

[51] Freeborn K, Portillo CJ (2017). Does pre-exposure prophylaxis (PrEP) for HIV prevention in men who have sex with men (MSM) change risk behavior? A systematic review. J Clin Nurs.

[52] Jenness SM, Sharma A, Goodreau SM, Rosenberg ES, Weiss KM, Hoover KW, Smith DK, Sullivan P (2017). Individual HIV risk versus population impact of risk compensation after HIV preexposure prophylaxis initiation among men who have sex with men. PloS One.

[53] Klevens RM, Martin BM, Doherty R, Fukuda HD, Cranston K, DeMaria Jr A (2017). Factors associated with pre-exposure prophylaxis in a highly insured population of urban men who have sex with men. AIDS Behav.

[54] Merchant RC, Corner D, Garza E, Guan W, Mayer KH, Brown L, Chan PA (2016). Preferences for HIV pre-exposure prophylaxis (PrEP) information among men-who-have-sex-with-men (MSM) at community outreach settings. J Gay Lesbian Ment Health.

[55] Okafor CN, Gorbach PM, Ragsdale A, Quinn B, Shoptaw S (2017). Correlates of preexposure prophylaxis (PrEP) use among men who have sex with men (MSM) in Los Angeles, California. J Urban Health.

[56] Hubach RD, Currin JM, Sanders CA, Durham AR, Kavanaugh KE, Wheeler DL, Croff JM (2017). Barriers to access and adoption of pre-exposure prophylaxis for the prevention of HIV among men who have sex with men (MSM) in a relatively rural state. AIDS Educ Prev.

[57] Arnold T, Brinkley-Rubinstein L, Chan PA, Perez-Brumer A, Bologna ES, Beauchamps L, Johnson K, Mena L, Nunn A (2017). Social, structural, behavioral and clinical factors influencing retention in Pre-Exposure Prophylaxis (PrEP) care in Mississippi. PLoS One.

[58] Snowden JM, Chen YH, McFarland W, Raymond HF (2017). Prevalence and characteristics of users of pre-exposure prophylaxis (PrEP) among men who have sex with men, San Francisco, 2014 in a cross-sectional survey: implications for disparities. Sex Transm Infect.

[59] Juusola JL, Brandeau ML, Owens DK, Bendavid E (2012). The cost-effectiveness of preexposure prophylaxis for HIV prevention in the United States in men who have sex with men. Ann Intern Med.

[60] Saberi P, Gamarel KE, Neilands TB, Comfort M, Sheon N, Darbes LA, Johnson MO (2012). Ambiguity, ambivalence, and apprehensions of taking HIV-1 pre-exposure prophylaxis among male couples in San Francisco: a mixed methods study. PLoS One.

[61] Hood JE, Buskin SE, Dombrowski JC, Kern DA, Barash EA, Katzi DA, Golden MR (2016). Dramatic increase in preexposure prophylaxis use among MSM in Washington state. AIDS.

